# Control of Bacteraemia from Indwelling Central Venous Catheters

**Published:** 1988

**Authors:** N. C. Weightman

**Affiliations:** Bristol Royal Infirmary


Bristol Medico-Chirurgical Journal Special Supplement 102 (1a) 1988

### Control of bacteraemias from indwelling central venous catheters

**N. C. Weightman**

Bristol Royal Infirmary

Since the introduction of indwelling central venous catheters by Broviac (1) and Hickman (2), their use has become widespread in children requiring chemotherapy for malignant disease. A major complication of their use is infection (3), particularly bacteraemia related to colonisation of the catheter.

There are two aspects of control of catheter-related bacteraemias; firstly, their prevention and secondly, their treatment. In order that these aspects can be adequately investigated and assessed, it is necessary to be able to make a firm diagnosis that a bacteraemia is catheter-related.

Several definitions of catheter-related bacteraemia have been used, including resolution of fever and bacteraemia upon removal of a catheter (4), semiquantitative culture of removed catheter tips (5), and all bacteraemias of undetermined origin (6). The former two definitions preclude attempts to eradicate infection without catheter removal, and the latter will include those bacteraemias with no obvious clinical source which are known to occur in patients with malignant disease and no catheters (7). Quantitative blood cultures have been successfully used to diagnose catheter-related bacteraemia (8), obviating the need for catheter-removal. A technique involving direct inoculation of blood on to agar has been described (9), high colony counts in samples taken through the catheter relative to those in samples from peripheral blood implicating the catheter as the source of the bacteraemia.

With the ability to diagnose catheter-related bacteraemia, its pathogenesis can be investigated and an understanding of the source of these infections may lead to preventative measures being taken. There is however debate over the pathogenesis of catheter-related bacteraemia. The flora at the catheter insertion site has been suggested as the source of infection in catheters used for total parenteral nutrition (10), although this work has rightly been criticised (11). Contamination of the catheter hub leading to colonisation of the lumen of the catheter has more recently been implicated (12,13). Prevention of this contamination might be expected to lead to a reduction in catheter-related bacteraemia.

Once infected, it has been suggested that intravenous catheters should be removed (14). However, with Hickman catheters this procedure is not without risk, particularly in immunocompromised thrombocytopaenic children. The possibility of eradicating such infections by appropriate bactericidal antibiotics given intravenously through the catheter has been demonstrated (15,16).

To investigate the pathogenesis, diagnosis, treatment and possible prevention of catheter-related bacteraemias, a one year prospective study of infections in 41 children with Hickman catheters was undertaken at the Bristol Children's Hospital. Quantitative blood cultures were used, and proved to be a reliable and economical method of diagnosing catheter-related bacteraemia. Culture of catheter insertion sites and catheter hubs in a semiquantitative manner suggested that contamination of the catheter hub is the initial step in many cases of catheter-related bacteraemia, 80% of cultured hubs being colonized with the same organism as that causing the catheter-related bacteraemia. Attempts at treating catheter-related bacteraemias without removing the catheter were made, and were successful in all 9 episodes that could be assessed. A fall in incidence of catheter-related bacteraemia, from 5.8 to 2.5 per 1000 catheter days use, following a change in the protocol for care of the catheters, with a more strict aseptic technique and reduced frequency of manipulation suggests that catheter-related bacteraemia may be controlled in terms of prevention, as well as treatment.

### REFERENCES

1. BROVIAC, J. W., COLE, J. J., SCRIBNER, B. H., (1973) A silicone rubber atrial catheter for prolonged parenteral alimentation. *Surg.Gynecol.Obstet* **136**, 602–6.
2. HICKMAN, R. O., BUCKNER, C. D., CLIFT, R. A., SANDERS, J. E., STEWART, P., THOMAS, E. D. (1979) A modified right atrial catheter for access to the venous system in marrow transplant recipients. *Surg.Gynecol.Obstet.* **148**, 871–5.
3. EDITORIAL. (1985) Indwelling venous catheters. *Lancet* **i**, 499.
4. PRESS, O. W., RAMSEY, P. G., LARSON, E. B., FEFER, A., HICKMAN, R. O. (1984) Hickman catheter infections in patients with malignancies. *Medicine (Baltimore)* **63**, 189–200.
5. MAKI, D. G., WEISE, C. E., SARAFIN, H. W. (1977) A semiquantitative culture method for identifying intravenous-catheter-related infection. *New.Engl.J.Med.* **296**, 1305–9.
6. SHAPIRO, E. D., WALD, E. R., NELSON, K. A., SPIEGELMAN, K. N. (1982) Broviac catheter-related bacteraemia in oncology patients. *Am.J.Dis.Child.* **136**, 679–681.
7. KETCHEL, S. J., RODRIGUEZ, V. (1978) Acute infections in cancer patients. *Semin.Oncol.* **5**, 167–179.
8. WEIGHTMAN, N. C., SPELLER, D. C. E. (1986) Pour plate blood cultures to detect bacteraemias related to indwelling central venous catheters. *J.Hosp.Inf.* **8**, 203–204.
9. RAUCHER, H. S., HYATT, A. C., BARZILAI, A., HARRIS, M. B., WEINER, M. A., LELEIKO, N. S., HODES, D. S. (1984) Quantitative blood cultures in the evaluation of septicemia in children with Broviac catheters. *J.Paediatr* **104**, 29–33.
10. SNYDMAN, D. R., GORBEA, H. F., POBER, B. R., MAJKA, J. A., MURRAY, S. A., PERRY, L. K. (1982) Predictive value of surveillance skin cultures in total-parenteral-nutrition-related infection. *Lancet* **ii**, 1385–1388.
11. SITGES-SERRA, A., JAURIETTA, E., LINARES, J., PEREZ, J. L., GARAU, J. (1983) Bacteria in total parenteral nutrition catheters: where do they come from? *Lancet* **i**, 531.
12. LINARES, J., SITGES-SERRA, A., GARAU, J., PEREZ, J. L., MARTIN, R. (1985) Pathogenesis of catheter sepsis: a prospective study with quantitative and semiquantitative cultures of catheter hub and segments. *J.Clin.Micro.* **21**, 357–360.
13. WEIGHTMAN, N. C., SIMPSON, E. M., SPELLER, D. C. E. (1986) Source of infection in Hickman catheters. *J.Clin.Path.* **39**, 1046.
14. EYKYN, S. J. (1984) Infection and intravenous catheters. *J.Antimicrob.Chemother.* **14**, 203–208.
15. DARBYSHIRE, P. J., WEIGHTMAN, N. C., SPELLER, D. C. E. (1985) Problems associated with indwelling central venous catheters. *Arch.Dis.Chil.* **60**, 129–134.

